# CaMKII and stress mix it up in mitochondria

**DOI:** 10.3389/fphar.2014.00067

**Published:** 2014-05-01

**Authors:** Mei-ling A. Joiner, Olha M. Koval

**Affiliations:** Internal Medicine/Cardiology, University of IowaIowa City, IA, USA

**Keywords:** mitochondria, cell death, mitochondrial calcium uniporter, CaMKII, CaMKIIN

## Abstract

CaMKII is a newly discovered resident of mitochondria in the heart. Mitochondrial CaMKII promotes poor outcomes after heart injury from a number of pathological conditions, including myocardial infarction (MI), ischemia reperfusion (IR), and stress from catecholamine stimulation. A study using the inhibitor of CaMKII, CaMKIIN, with expression delimited to myocardial mitochondria, indicates that an underlying cause of heart disease results from the opening of the mitochondrial permeability transition pore (mPTP). Evidence from electrophysiological and other experiments show that CaMKII inhibition likely suppresses mPTP opening by reducing Ca^2+^ entry into mitochondria. However, we expect other proteins involved in Ca^2+^ signaling in the mitochondria are affected with CaMKII inhibition. Several outstanding questions remain for CaMKII signaling in heart mitochondria. Most importantly, how does CaMKII, without the recognized N-terminal mitochondrial targeting sequence transfer to mitochondria?

## Introduction

**Figure 1:**
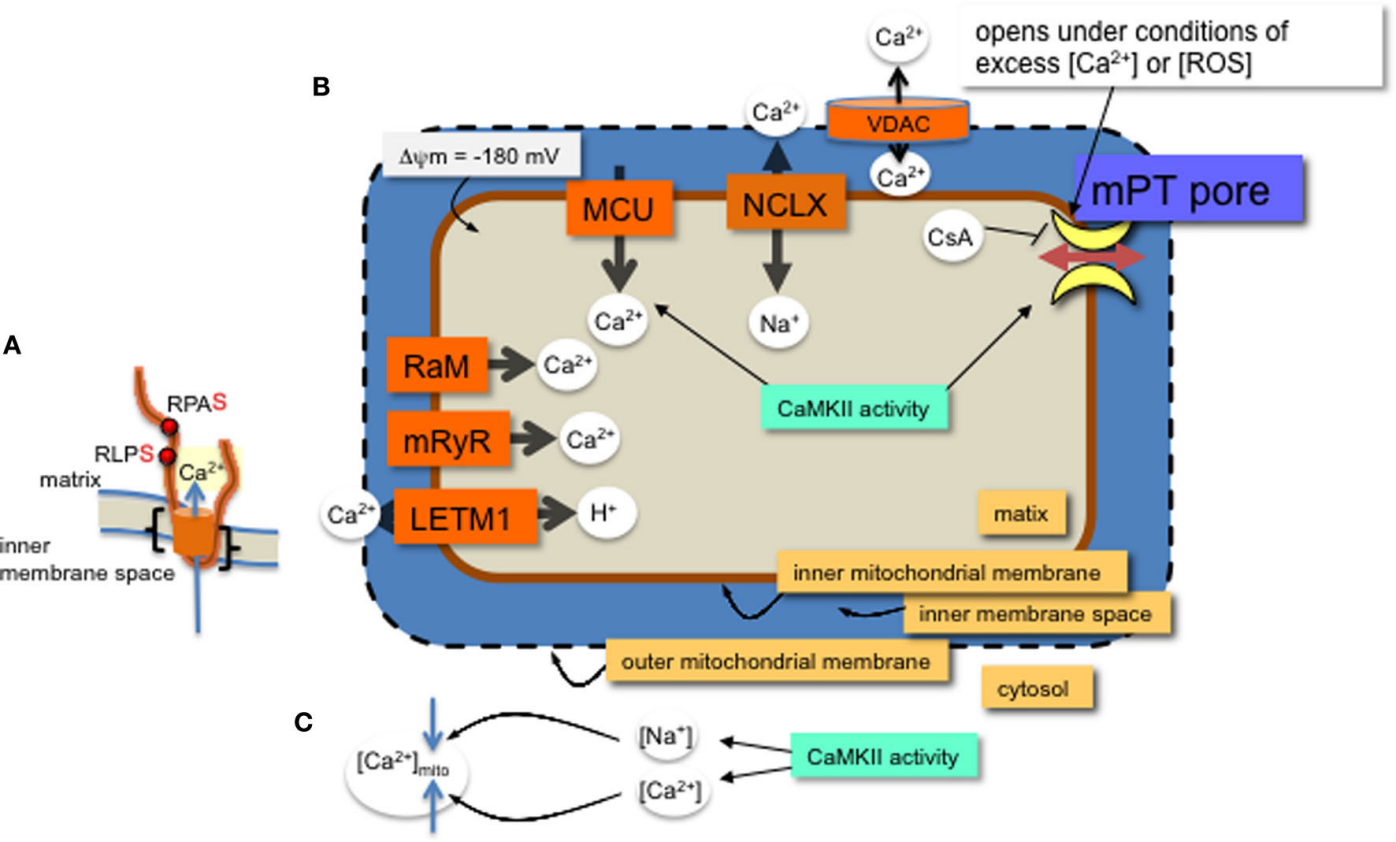
**MCU channel in the inner mitochondrial membrane. (A)** A single monomer of MCU is shown (orange) with two phospho-serine residues (red dots, with consensus amino acids adjacent) on the N-terminal region in the matrix. The two transmembrane domains are indicated (black brackets). Layers of regulation include accessory proteins and a protein similar to MCU, MCUb (not shown). **(B)** Mitochondrial Ca^2+^ channels and exchangers on the inner membrane. Ca^2+^ predominantly enters the matrix through the MCU channel and efflux is via the Na^+^-Ca^2+^ exchanger (NCLX). Other channels and an exchanger that were found to regulate Ca^2+^ across the inner membrane are the rapid mode of uptake (RaM), the ryanodine receptor (mRyR) and the Ca^2+^-H^+^ exchanger (LETM1). Voltage dependent anion channels (VDAC) allow ions and metabolites across the outer membrane. The proton (H^+^) gradient, a major component of the membrane potential (ΔΨ), is generated from the electron transport chain and drives the flow of H^+^ through ATP synthase in a reaction coupled to the generation of ATP from ADP and inorganic phosphate. The membrane potential produces a driving force for matrix Ca^2+^ accumulation. Excess Ca^2+^ or ROS will open the permeability transition pore, which can be inhibited with CsA or CaMKIIN. Mitochondrial CaMKII activity regulates 1. Ca^2+^ entry through the MCU and 2. transition pore opening. **(C)** CaMKII activity in the cytosol can increase both Ca^2+^ and Na^+^ ion levels in the cytosol with opposite effects on mitochondrial matrix Ca^2+^ accumulation.

CaMKII activity promotes heart failure by mediating pathological effects of ischemia reperfusion (IR) through induction of both apoptosis and necrosis (Salas et al., 2009). Cytosolic inhibition of CaMKII attenuates cell death in the heart that results from catecholamine stress, myocardial infarction (MI) or IR (Yang et al., 2006). The increase in cell death via CaMKII activity involves mitochondrial pro-death pathways (Salas et al., 2009; Joiner et al., 2012). Further, either membrane partitioned or mitochondrial matrix expression of a specific and potent inhibitor of CaMKII, CaMKIIN, reduces cell death from MI, catecholamine stress, and IR (Joiner et al., 2012). Therefore, inhibiting CaMKII either in the cytosol or in mitochondria can block CaMKII activity leading to cell death. CaMKII protein targets in cytosol are well studied and include Ca^2+^ entry pathways and proteins involved with Ca^2+^ handling at the ER (Salas et al., 2009; Koval et al., 2010; Ozcan and Tabas, 2010; Zhang et al., 2010). Mitochondrial-triggered cell death occurs from Ca^2+^ overload or excess reactive oxygen species (ROS) production in the mitochondria (Crompton and Costi, 1988; Gunter and Pfeiffer, 1990; Lemasters et al., 2009). Inhibiting or eliminating mitochondrial CaMKII activity reduced cell death in a number of cellular models of pathology (Timmins et al., 2009; Joiner et al., 2012; Yun et al., 2013). Reducing cell death by mitochondria Ca^2+^ overload may occur by either decreasing Ca^2+^ uptake or reducing mitochondrial permeability transition pore (mPTP) formation (Griffiths and Halestrap, 1993; Elrod et al., 2010; Pan et al., 2013) (Figure 1). The predominant mitochondrial Ca^2+^ uptake and efflux are via the mitochondrial calcium uniporter (MCU) and Na^+^/Ca^2+^ antiporters (NCLX), respectively. However, a number of other channels have been described for these processes (Figure 1 and described in recent reviews, including Ryu et al., 2010). Regulation of these ion channel complexes by post-translational modification and auxiliary proteins is best described for the MCU. In addition to Ca^2+^ exchange through channel proteins, Ca^2+^ can be sequestered in the matrix by forming phosphate complexes. These Ca^2+^-phosphate complexes allow accumulation of Ca^2+^ in the matrix during periods of high levels cytosolic Ca^2+^ (Wei et al., 2012). In this review we focus on effects of CaMKII in mitochondrial Ca^2+^ uptake and permeability transition.

## CaMKII effects on MCU

Mitochondria take up Ca^2+^ primarily via the MCU (Kirichok et al., 2004; Baughman et al., 2011; De Stefani et al., 2011). The MCU pore-forming channel, a 350 amino acid protein, has two predicted transmembrane helices (Figure 1, from amino acids Lys233 to Trp255 and Thr266 to Met 283), each spanning the inner mitochondrial membrane with the N- and C-terminal ends extending into the matrix. The MCU channel is composed of two pore forming proteins, MCUa and MCUb, as well as at least three regulatory proteins, MICU1, MICU2, and EMRE (Perocchi et al., 2010; Sancak et al., 2013). Although a number of post-translational modifications were identified for MICU1 (Hornbeck et al., 2012), it is not known how these affect Ca^2+^ current or whether CaMKII phosphorylates any of the accessory proteins or MCUb.

A number of research groups use patch clamp onto mitoplasts (exposed inner membrane of mitochondrial) to measure ion currents, such as the MCU current, across the inner mitochondrial membrane. In order for the patch pipette to access the mitochondrial inner membrane, the mitochondria must be swollen to rupture the outer mitochondrial membrane. These manipulations raise the issue of whether, or how closely, the channel activity observed with patch-clamp corresponds to the *in vivo* state. Technical aspects of mitoplast patch-clamping remain highly non-standardized, as evidenced by inter-study variation in mitoplast capacitance (0.8–5 pF), (Kirichok et al., 2004; Fieni et al., 2012; Chaudhuri et al., 2013; Dolga et al., 2013; Hoffman et al., 2013) and patch-electrode resistance (4–40 MΩ) (Kirichok et al., 2004; Dolga et al., 2013). In combination with these technical aspects is the broader uncertainty in basic physiological characteristics of mitochondria, such as the ionic composition of the matrix. Reduced experimental conditions along with technical issues for patch-clamp studies of mitochondrial inner membrane ion currents can complicate data interpretation. For example, theoretical estimates of ionic currents are orders of magnitude lower than the currents through mitochondrial channels directly measured by patch-clamp (Kane and Pavlov, 2013). Accordingly, to understand channel modifications and regulation, studies should include a variety of techniques, not only patch clamp. Despite these acknowledged variations, overall effects of manipulating CaMKII function or its target sites in MCU have revealed effects of CaMKII activation on mitochondrial Ca^2+^ uptake. That is, phosphorylation of two serine sites on the N-terminus of MCU present a phenotype when mutated. Significantly, the CaMKII-induced larger current can be prevented by serine to alanine mutations of these two residues on MCU (Joiner et al., 2012) (Figure 1).

In order to identify proteins in the mitochondrial CaMKII pathway, we deduced that MCU may be a target for CaMKII because well-established CaMKII targets in heart cells are located near Ca^2+^ sources (Koval et al., 2010; Purohit et al., 2013). Furthermore, recent publications that showed either CaMKII inhibition (Yang et al., 2006) or the MCU inhibitor, Ru360 (García-Rivas Gde et al., 2006) are protective from IR damage in heart. An immunoprecipitation assay indicated that mitochondrial CaMKII and the MCU interact in a complex (Joiner et al., 2012) and others showed that accumulation of mitochondrial Ca^2+^ is activated through CaMKII signaling (Timmins et al., 2009). Ca^2+^ current through the MCU is increased with CaMKII activation as shown with patch-clamp measurements onto prepared mitoplasts and expression of CaMKIIN in the matrix reduced mitochondrial Ca^2+^ uptake (Joiner et al., 2012). Others found that CaMKII inhibitors block A23187-stimulated arachidonic acid release, LDH release and the decrease in the subsequent mPTP formation is attributed to reduced MCU current (Yun et al., 2013). Taken together, these studies indicate that CaMKII activation in mitochondria is responsible for excess Ca^2+^ uptake under pathological conditions, which ultimately leads to increased levels of cell death. Conversely, in the absence of MCU, using a knock out mouse lacking the MCU channel, Pan et al showed no protective effect preventing necrosis in the heart after IR (Pan et al., 2013). This study suggests that phosphorylation by CaMKII of a protein(s), other than on the MCU, underlies the transition to cell death by CaMKII activation.

## CaMKII effects on mPTP

Inhibiting mitochondrial CaMKII with CaMKIIN expression decreased cell death following MI, catecholamine stress and IR. As suggested above, phosphorylation of the MCU by CaMKII may promote cell death under stress conditions. However, reducing MCU current does not appear effective in reducing apoptosis (Pan et al., 2013), therefore, an alternative pathway for CaMKII inhibition may be to delay mPTP opening. The mitochondrial permeability transition allows flow of molecules of up to 1500 Daltons to pass across the inner mitochondrial membrane, leading to mitochondrial swelling, and cell death through apoptosis or necrosis. Opening of the transition pore on the inner membrane occurs under pathophysiological conditions and is triggered by either excess Ca^2+^ or ROS (Kim et al., 2006; Lemasters et al., 2009). The ATP synthase complex on the inner membrane is a leading contender for molecular identity of the transition pore (Giorgio et al., 2013). In addition, the phosphate carrier and auxiliary regulatory proteins are possible components (Halestrap, 2009). Blocking the opening of the mPTP with the inhibitor, cyclosporin A (CsA) (Nicolli et al., 1996; Halestrap and Brenner, 2003) can reduce cell death from stress and reduce infarct size in hearts after IR in patients (Piot et al., 2008). A number of mitochondrial kinases appear to regulate mPTP opening (Miura et al., 2010; Azarashvili et al., 2014). Furthermore, like CsA, CaMKII inhibition increases matrix Ca^2+^ capacity because expressing CaMKIIN in the mitochondrial matrix allowed as much or more Ca^2+^ retention as did CsA (Joiner et al., 2012), suggesting a level of regulation by CaMKII and other kinases in transition pore formation.

## CaMKII effects on metabolism

Protective effects of CaMKII inhibition may occur via auxiliary proteins to the transition pore. CaMKII regulates the interaction of carnitine palmitoyltransferase I with its inhibitor, malonyl CoA, to affect fatty acid metabolism in mitochondria (Sharma et al., 2010) with possible consequences for mPTP opening (Moon et al., 2012). A study using a knockdown approach to decrease a number of kinases, including CaMKII, showed decreases in ATP synthase activity correlated with a reduction in kinase activity (Sugawara et al., 2013). As mentioned above, components of the ATP synthase complex may form the transition pore under stress conditions (Giorgio et al., 2013). Taken together, excess CaMKII activation may promote mitochondrial cell death by its link to energy production and transition pore formation.

## CaMKII targets identified by mitochondrial phosphoproteomics

Many post-translational modifications are being discovered in the mitochondrial proteome that are yet to be established as functionally significant. Using phosphoproteomics is one way to identify potential CaMKII targets in the mitochondria. However, it has been argued that few transient protein phosphorylation events are physiologically relevant (Clarke et al., 2008; Covian and Balaban, 2012), citing few phospho-sites revealed with a phospho-protein fluorescent dye on mitochondrial lysate before and after treatment to induce mPTP inhibition (Clarke et al., 2008), and also reasoning that phosphorylation can occur spontaneously, without a kinase. In contrast, numerous sensitive phosphoproteomic studies have identified hundreds of phospho-sites on mitochondrial proteins under different treatment regimes (Lee et al., 2007; Witze et al., 2007; Zhao et al., 2011; Koc and Koc, 2012), some of which are indeed functionally relevant as described in the previous two sections. The spontaneous reaction argument is reminiscent of the early days of assigning a role to superoxide dismutase. Arguments that an enzyme for the superoxide radical dismutation to H_2_O_2_ was not necessary, as the reaction could occur rapidly without an enzyme (Fridovich, 1983), were eventually overruled by findings that superoxide dismutase over expression or reduced expression can lead to drastic physiological changes *in vivo* (Antonarakis et al., 2004). Ultimately, uncovering functionally relevant phospho-sites, for CaMKII and other kinases, in the mitochondrial proteome will require extensive study of the individual sites under different phosphorylation conditions along with mutation analysis.

## Specificity of mitochondrial CaMKII phosphorylation sites

Protein phosphorylation sites identified by phosphoproteomics described above may be attributed to kinases other than CaMKII. The consensus phosphorylation site, a serine or threonine, three amino acids down stream of an arginine (RxxS/T, x represents any amino acid) for CaMKII phosphorylation is shared by a number of other kinases, for example, PKCdelta (www.kinexus.ca). However, specificity of the inhibitor CaMKIIN occurs even in overexpression systems, where for example both CaMKII and PKC could phosphorylate the same consensus site. When each kinase was co-expressed with CaMKIIN, only phosphorylation by CaMKII was inhibited (Chang et al., 1998). Therefore, using the CaMKII inhibitor, CaMKIIN, endogenous to brain (Chang et al., 1998) raises confidence that CaMKII, rather than a different kinase, is responsible for promoting cell death under stress conditions in heart.

## Impact of non-mitochondrial CaMKII on mitochondrial function in disease

CaMKII activity outside of mitochondria contributes to mitochondrial Ca^2+^ homeostasis. CaMKII activity elevates diastolic sarcoplasmic reticulum (SR) Ca^2+^ leak (Curran et al., 2007), which was later shown to contribute to mitochondrial Ca^2+^ overload (Zhang et al., 2010) specifically, under pathophysiological conditions such as rapid cardiomyocyte pacing (Sepúlveda et al., 2013) and diabetes (Luo et al., 2013), but also with the extreme physiological condition of endurance exercise (Rose et al., 2007). Disruption of cytosolic Ca^2+^ homeostasis promotes mitochondrial Ca^2+^ overload (Lemasters et al., 2009). Using genetic tools to overexpress CaMKII or the inhibitor of CaMKII, CaMKIIN, in different cell compartments will lead to a better understanding of where CaMKII activity is required for promoting disease with particular models of stress, including MI, IR, excess catecholamine stimulation, and metabolic diseases. Conversely, CaMKII is a major contributor to myocyte Na^+^ homeostasis in heart failure (Wagner et al., 2006) and Na^+^ accumulation in heart failure was shown to influence mitochondrial Ca^2+^ load via enhanced NCLX-mediated Ca^2+^ removal (Maack et al., 2006). Thus, indirectly, cytosolic CaMKII can regulate mitochondrial Ca^2+^ levels.

## Summary

The role of CaMKII functioning in the mitochondria in physiology and disease is in the early stages of research and discovery. CaMKII may be central to regulating mitochondrial homeostasis as its activity is regulated by both Ca^2+^ (Miller and Kennedy, 1986) and ROS (Erickson et al., 2008) signaling pathways. CaMKII effects in the mitochondria are likely to be numerous and uncovering target sites promises to reveal regulation of mitochondrial signaling pathways that tune cellular responses for cardiac output.

### Conflict of interest statement

The authors declare that the research was conducted in the absence of any commercial or financial relationships that could be construed as a potential conflict of interest.
